# Mobile Pantries Can Serve the Most Food Insecure Populations

**DOI:** 10.1089/heq.2021.0006

**Published:** 2022-01-24

**Authors:** Lily K. Villa, Shakthi Bharathi Murugesan, Lora A. Phillips, Alexandria J. Drake, Nathan A. Smith

**Affiliations:** ^1^ School of Human Evolution and Social Change, Arizona State University, Tempe, Arizona, USA.; ^2^ MAS Geographic Information Systems, School of Geographic Sciences and Urban Planning, Arizona State University, Tempe, Arizona, USA.; ^3^ Knowledge Exchange for Resilience, School of Geographic Sciences and Urban Planning, Arizona State University, Tempe, Arizona, USA.; ^4^ Phoenix Rescue Mission, Phoenix, Arizona, USA.

**Keywords:** food insecurity, mobile pantries, elderly, immigrant, Maricopa County, food deserts, assistance deserts, Food Systems Mapping

## Abstract

**Purpose:** Food insecurity is an urgent crisis in the United States, with one in nine people lacking a consistent source of the food necessary for an active and healthy lifestyle. This crisis is particularly dire in Maricopa County, Arizona, where 1 in 5 children experience food insecurity, and >1 in 10 residents experience poverty. Mobile food pantries offer an additional resource to address food insecurity; however, there is minimal knowledge about how communities utilize these food distributors.

**Background:** Research on the elderly (people >60 years) and immigrant populations shows that these populations are especially vulnerable to food insecurity. The risk these groups face is compounded in Maricopa County, the 15th largest county in the country with minimal public transit to extant resources. Mobile food pantries offer one solution to this issue, bringing groceries and other important items directly to communities.

**Methods:** This study utilizes data from a food pantry called “Phoenix Rescue Mission” (PRM) on food insecure people's use of PRM's mobile and brick-and-mortar pantries, as well as census data. Using GIS mapping and a multinomial logistic regression model, this research identifies how different demographic groups engage with PRM's brick-and-mortar or mobile pantries.

**Results:** Findings indicate that people aged 60–80 years and immigrant people of color are more likely to use both mobile and brick-and-mortar pantries.

**Conclusions:** This research suggests that mobile pantries can reach the most food insecure populations and local nonprofits and governments can consider implementing mobile pantries to reach food insecure communities.

## Introduction

Food insecurity is an urgent crisis in the United States, with one in nine people lacking a consistent source of the food necessary for an active and healthy lifestyle.^1^ This crisis is particularly dire in Maricopa County, Arizona, where 1 in 5 children experience food insecurity,^2^ and >1 in 10 residents experience poverty.^3^ Individuals most vulnerable to food insecurity include ethnic and racial minorities,^4^ immigrants,^5^ the elderly,^6^ and people who are underemployed or unemployed.^7^

Food insecurity is associated with malnutrition^8^ and obesity^9^ and can potentially contribute to chronic conditions, such as cardiovascular disease^10^ and diabetes.^11^ In response to this public health crisis, many nonprofit organizations developed food pantries,^12^ farmer's markets,^13^ and food delivery services^14^ to mitigate the impacts of food insecurity.

In Maricopa County alone, >100 free food distribution services exist.^15^ Although mobile food pantries offer an additional resource to address food insecurity, there is minimal knowledge about how community members use this food distribution option. This research uses data from Phoenix Rescue Mission (PRM), a Maricopa County nonprofit with mobile and brick-and-mortar pantries, to understand what demographics of people use mobile pantries.

Some of the people most vulnerable to food insecurity are the elderly (adults aged 60–80 years).^16^ One in five Americans are aged 60+^17^ and the number of food insecure seniors has been rising since 2017.^16^ Among seniors, Hispanics and African Americans are 2–3 times more likely to be food insecure than white seniors.^16^ In Maricopa County, seniors make up ∼15% of the population who live at or below the poverty line.^18^

Senior food insecurity is linked to physical limitations such as arthritis, joint pain, poor physical function, and weight-related disability.^19^ To address senior food insecurity, many nonprofits in Maricopa County offer meals on wheels food delivery services, Commodity Supplemental Food Programs, and senior centers with free cafeterias. Owing to closures during the coronavirus disease 2019 (COVID-19) pandemic, many senior centers and food distributors have transitioned to drive-through and mobile pantry services, but little is known about how effective these services are for addressing senior food insecurity.

In addition to seniors, immigrant populations are particularly vulnerable to food insecurity. Extant research asserts that ethnic and racial minorities are some of the most food insecure people in the United States due to high levels of underemployment and limited access to immigration documentation.^20^ In Arizona, two out of five immigrant households are low income.^21^ Local-level immigration enforcement policies that reduce non-U.S. citizens' access to social services contribute to these disparities.^5^ Moreover, many low-income seniors^22^ and immigrants^23^ live in food deserts, which exacerbates their food insecurity.

Food deserts are areas with limited or no access to fresh food within walking distance,^24^ and “assistance deserts” are areas where food pantries are more than a mile from people's homes.^25^ Maricopa County is the 15th largest county by area in the United States with a sprawling mix of urban, suburban, and rural areas.^2^

Consequently, the average resident must commute 26 min to their place of work,^3^ suggesting that most Maricopa County residents are heavily dependent upon personal vehicles. Moreover, food deserts and assistance deserts pepper the Maricopa County landscape with many people living more than a mile from a grocery store^26^ or food pantry.^27^ However, many low-income immigrant families and senior citizens have limited access to private transportation to and from local food pantries.^28^ One way to counteract food insecurity in food and assistance deserts is to offer mobile food pantries, which many nonprofits have provided in low-income areas throughout the United States.^26^

How effective are mobile pantries in mitigating food insecurity? Our research seeks to identify how different demographic groups engage with brick-and-mortar or mobile pantries, using PRM's data coupled with census data. An increased understanding of pantry use patterns allows for more targeted interventions, food pantry programming, and outreach. This article addresses the following questions: Do mobile food pantries serve a distinct set of clients relative to brick-and-mortar food pantries? And are some clients using both? If so, how do the (1) demographic and (2) economic characteristics of clients differ across service distribution types?

## Materials and Methods

After obtaining Institutional Review Board approval for this research, we leveraged de-identified quantitative data provided by PRM. When people first visit one of the PRM food pantries, they complete an extensive intake questionnaire administered by a trained PRM employee or volunteer. This intake questionnaire includes detailed information on clients' demographic and economic background, as well as their residence location. Those clients who return for future visits also go through the intake process to confirm that their information has not changed, thus providing detailed information on when and where clients move through the PRM food pantry system.

The data constitute 9803 unique clients, with 9477 clients visiting a brick-and-mortar pantry, 174 visiting a mobile pantry, and 152 visiting both pantry types, over 9 months beginning in October 2019. (These figures do not include 30 clients who were dropped due to missing data on pantry type due or misentered data on income—e.g., negative values—all of whom were clients of the brick-and-mortar pantry).

Using a multinomial logistic regression, where the dependent variable is a categorical indicator of brick-and-mortar client (reference group), mobile-only client, or brick-and-mortar and mobile client, we regress age, household size, monthly gross income, gender, marital status, and race/ethnicity. Age, household size, and monthly gross income are continuous. Gender is a categorical indicator of male (reference group), female, or other. Marital status is a categorical indicator of single (reference group), married or common law marriage, separated or divorced, or widowed.

Finally, race/ethnicity is a categorical indicator of non-Hispanic white alone (reference group), non-Hispanic American Indian alone; non-Hispanic Asian alone; non-Hispanic black alone; non-Hispanic Middle-Eastern or North African alone; Hispanic alone; other; and undisclosed. These racial/ethnic categories were selected due to population coverage across the different pantry usage types. These data were supplemented with zip code-level data from the American Communities Survey (5-year estimates, 2014–2018).

PRM is located in Phoenix, Arizona and their mission is to be a “leading provider of Christ-centered life-transforming solutions to persons facing hunger, homelessness, addiction, and trauma.”^29^

PRM offers a holistic approach to addressing their clients' needs, such as providing food, water, shelter, hygiene products, and clothing. PRM's “Hope for Hunger” food pantry and hygiene product distribution center is a brick-and-mortar pantry located in Glendale, Arizona.

Hope for Hunger offers their services to people throughout Maricopa County Monday–Friday 8 am–12 pm. To serve people in food and assistance deserts in Maricopa County, PRM also provides the mobile “Peoria Mobile Food Pantry” and a mobile Saturday Market, which offer their services in the central and south city of Phoenix on the second and fourth Saturday each month.

PRM additionally offers the “Murphy Mobile Pantry” that is open the first Thursday of the month in central Phoenix. These pantries are extensions of the brick-and-mortar pantry and provide temporary “pop-up” distribution centers. Although they are not mobile in the sense that they return to the same churches and community centers and remain parked for food distribution, they are mobile in the sense that the pantries are vans that can easily change locations to meet their clients' needs. PRM selects these sites based upon voting district poverty statistics, placing the pantries in some of the most food insecure assistance deserts in the Phoenix area.

**FIG. 1.**
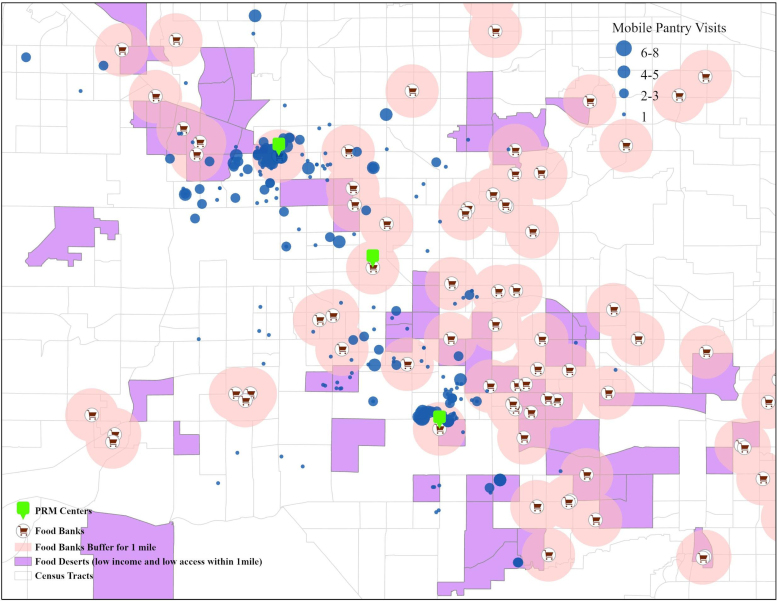
Map of food pantry locations as they relate to food deserts.

Figure 1 displays the location of PRM food pantries, the number of times that clients visited these pantries over 9 months, and the location of food deserts and food assistance deserts in Maricopa County. Food deserts, defined by the United States Department of Agriculture (USDA) as low-income and low-access tracts measured at 1.6 Km for urban areas and 10 miles for rural areas, are mapped. Assistance deserts in this context are defined as a 1-mile radius around food pantries. The areas outside the 1-mile radii are other assistance deserts, but may or may not be food deserts.

## Results

Table 1 shows the regression results. The odds of a client going to a mobile pantry versus a brick-and-mortar pantry increase by 63.6% when the client is female as compared with male, holding other variables constant (*p*<0.05). In terms of race/ethnicity, the odds of a client going to a mobile pantry versus a brick-and-mortar pantry increase by 198.8% and 126.1%, respectively, when the client is Middle Eastern/North African or Hispanic as compared with white, holding other variables constant (*p*<0.01, *p*<0.001). Although income is also a significant predictor of mobile pantry usage (*p*<0.001), the coefficient makes clear that this predictor is statistically but not substantively significant.

**Table 1.** Multinomial Logistic Regression Results

Service type	Variable	Coef. (SE) (base outcome)
Brick-and-mortar only		
Mobile only	Age	0.009 (0.006)
	Household size	−0.082 (0.045)
	**Female**	0.492^*^ (0.193)
	Other gender	1.416 (1.061)
	Married and common law	0.280 (0.174)
	Divorced and separated	−0.178 (0.308)
	Widowed	0.408 (0.378)
	**Middle Eastern/North African**	1.095^**^ (0.401)
	American Indian	0.714 (0.620)
	Asian	0.548 (0.622)
	African American	−0.553 (0.456)
	**Hispanic**	0.816^***^ (0.225)
	Other race/ethnicity	0.782 (0.745)
	Undisclosed race/ethnicity	0.740 (0.548)
	**Monthly income**	0.000^**^ (0.000)
Brick-and-mortar and mobile	**Age**	0.031^***^ (0.006)
	Household size	0.038 (0.042)
	Female	0.035 (0.200)
	Other gender	−11.089 (424.85)
	**Married and common law**	0.645^**^ (0.374)
	Divorced and separated	0.006 (0.341)
	Widowed	0.656 (0.374)
	**Middle Eastern/North African**	1.842^***^ (0.419)
	**American Indian**	1.759^**^ (0.579)
	**Asian**	1.635^**^ (0.535)
	African American	0.143 (0.491)
	**Hispanic**	1.327^***^ (0.293)
	Other race/ethnicity	1.389 (0.769)
	Undisclosed race/ethnicity	0.884 (0.765)
	Monthly income	0.000 (0.000)

Bold variables are statistically significant.

^*^ *p*<0.05, ^**^p<0.01, ^***^p<0.001.

SE, standard error.

Among those clients who use both pantry types, the odds of a client going to both as opposed to brick-and-mortar alone increase by 3.1% for each additional year of age, holding other variables constant (*p*<0.001). For instance, the predicted probability of going to both pantry types for a 65-year-old who is average on all other characteristics is 0.018, whereas the predicted probability for a 24-year-old who is average on all other characteristics is 0.005. In addition, clients in married or common-law partnerships had 90.7% higher odds of using both pantry types as opposed to brick-and-mortar alone (*p*<0.01) relative to single clients, holding other variables constant. In terms of race/ethnicity, the odds of a client going to both pantry types versus a brick-and-mortar pantry alone increase by 53%, 480.8%, 413.2%, and 277%, respectively, when the client is Middle Eastern/North African, American Indian, Asian, or Hispanic as compared with white, holding other variables constant (*p*<0.001, *p*<0.01, *p*<0.01, *p*<0.001).

These analyses do not include controls for neighborhood sociodemographic characteristics because (as shown in Fig. 1) clients of PRM travel from throughout Maricopa County to obtain services. Nonetheless, although the zip codes where the pantries are located do vary sociodemographically to some extent, this variation is not likely driving the results.

For instance, the zip code where the brick-and-mortar pantry is located is racially/ethnically 24.1% white, 9.3% African American, and 61.4% Hispanic. Among clients who visited one of the two mobile pantries (whether or not they also visited the brick-and-mortar pantry), they were relatively equally likely to go to either the Murphy Mobile Pantry (56%) or the Peoria Mobile Food Pantry (44%). Averaging the racial/ethnic composition of these two zip codes, residents are 33.2% white, 4% African American, and 58.1% Hispanic—roughly similar, if not less racially/ethnically diverse, than the zip code where the brick-and-mortar pantry is located.

## Discussion and Conclusions

This study sought to understand if mobile pantries serve different demographics of people than brick-and-mortar pantries. Findings indicate that immigrants, people of color, and women are more likely to utilize mobile pantries than other populations in Maricopa County. In addition, seniors, married and common-law married people, and Black, Indigenous, and People of Color are more likely to use both a brick-and-mortar pantry and the mobile pantries.

These findings are consistent with extant literature suggesting that immigrant populations,^5^ women,^30^ seniors,^19^ unmarried,^31^ and nonwhite people^4^ are more likely to experience food insecurity than other populations.

This study augments literature showing that mobile pantries can effectively expand food distribution to highly vulnerable populations such as seniors and immigrant people of color. These findings are encouraging given that other studies have found that utilizing mobile pantries in conjunction with culturally relevant nutrition education can increase immigrant populations' fresh produce intake by minimizing the travel time and cost for acquiring healthy foods.^12^ Local governments and nonprofits can consider using mobile pantries to expand their community outreach and encourage healthy diets among vulnerable populations.

This research is limited by the fact that it uses data collected by one organization; thus, more research is needed to compare other mobile food services in Maricopa County. This research also shows that the COVID-19 pandemic has increased food insecurity in Maricopa County, but more research is needed to understand whether this factor influenced the results. Further research is also needed to understand how all mobile and brick-and-mortar food pantries in Maricopa County interact with one another and how they can more effectively assist people in food and assistance deserts.

Moreover, given that many people in Maricopa County are car dependent, future research could map drive-time for procuring food along with food and assistance deserts to better understand community food assistance needs. Nonetheless, these findings offer promising insight into how mobile pantries can effectively counteract food insecurity and provide an option for highly vulnerable populations and areas for accessing nutrient-rich foods. Finally, the overall sample of mobile food pantry users was substantially lower than brick and mortar users. Future research can continue to look at the impact of mobile food pantries with a larger sample of mobile pantry users.

## Abbreviations Used

COVID-19: coronavirus disease 2019
PRM: Phoenix Rescue Mission
SE: standard error
USDA: United States Department of Agriculture
